# Protein Remote Homology Detection Based on an Ensemble Learning Approach

**DOI:** 10.1155/2016/5813645

**Published:** 2016-05-08

**Authors:** Junjie Chen, Bingquan Liu, Dong Huang

**Affiliations:** ^1^School of Computer Science and Technology, Harbin Institute of Technology Shenzhen Graduate School, Shenzhen, Guangdong 518055, China; ^2^School of Computer Science and Technology, Harbin Institute of Technology, Harbin, Heilongjiang 150001, China; ^3^Key Laboratory of Network Oriented Intelligent Computation, Harbin Institute of Technology Shenzhen Graduate School, Shenzhen, Guangdong 518055, China

## Abstract

Protein remote homology detection is one of the central problems in bioinformatics. Although some computational methods have been proposed, the problem is still far from being solved. In this paper, an ensemble classifier for protein remote homology detection, called SVM-Ensemble, was proposed with a weighted voting strategy. SVM-Ensemble combined three basic classifiers based on different feature spaces, including Kmer, ACC, and SC-PseAAC. These features consider the characteristics of proteins from various perspectives, incorporating both the sequence composition and the sequence-order information along the protein sequences. Experimental results on a widely used benchmark dataset showed that the proposed SVM-Ensemble can obviously improve the predictive performance for the protein remote homology detection. Moreover, it achieved the best performance and outperformed other state-of-the-art methods.

## 1. Introduction

In computational biology, protein remote homology detection is the classification of proteins into structural and functional classes given their amino acid sequences, especially, with low sequence identities. Protein remote homology detection is a critical step for basic research and practical application, which can be applied to the protein 3D structure and function prediction [1, 2]. Although remote homology proteins have similar structures and functions, they lack easily detectable sequence similarities, because the protein structures are more conserved than protein sequences. When the protein sequence similarity is below 35% at the amino acid level, the alignment score usually falls into a twilight zone [3, 4]. Therefore, it is often a failure to detect protein remote homology by computational approaches only based on protein sequence features. To improve the specificity and sensitivity of the detection, we proposed an ensemble learning method, which can combine basic classifiers based on different feature spaces.

Up to now, many methods for protein remote homology detection have been proposed, which can be categorized into three groups [5]: pairwise alignment algorithms, generative models, and discriminative classifiers. Early computational approaches for protein remote homology detection are pairwise alignment methods, which detect sequence similarities between any given two protein sequences by using Needleman-Wunsch global alignment algorithm [6, 7] and Smith-Waterman local alignment algorithm [8]. Later, some trade-off methods were proposed so as to trade reduced accuracy for improved efficiency, such as BLAST [9] and FASTA [10]. PSI-BLAST [11] iteratively builds a probabilistic profile of a query sequence and therefore a more sensitive sequence comparison score can be calculated [12]. After pairwise alignment methods, the predictive accuracy was significantly improved by using the generative algorithms. Generative models were iteratively trained by using positive samples of a protein family or superfamily; for example, HHblits [13] generates a profile hidden Markov model (profile-HMM) [14, 15] from the query sequence and iteratively searches through a large database.

Currently the discriminative methods achieve the state-of-the-art performance [16–19]. Different from pairwise algorithm and generative methods, the discriminative methods can easily embed various characteristics of protein sequences and learn the information from both positive and negative samples in a given benchmark dataset. A key feature of discriminative method is that its input requires fixed length feature vectors. Therefore, some researchers proposed various feature vectors for protein representation. Some methods are based on sequence information, physical and chemical properties of proteins [20–22], or secondary structure information [23, 24], such as SVM-DR [25]. Some methods are based on kernel method, such as SVM-Pairwise [5], SVM-LA [26], motif kernel [27], mismatch [28], SW-PSSM [29], and profile kernel [30]. Later, the performance of discriminative approaches is further improved by Top-n-gram, because it can transform protein profiles into pseudo protein sequences, which contain the evolutionary information [31–33].

Although many discriminative methods for protein remote homology detection have been proposed based on various feature extracting techniques, there is no attempt to combine these methods using an ensemble learning method to improve predictive performance. An ensemble classifier [34, 35] is built by combining a set of basic classifiers in weighted voting strategy to give a final determination in classifying a query sample. Ensemble classifiers have achieved great success in many fields, including protein-protein interaction sites [36], protein fold pattern recognition [22, 37], tRNA detection [38, 39], microRNA identification [40–44], DNA binding protein identification [45], and eukaryotic protein subcellular location prediction [46], because they are able to learn a more expressive concept in classification compared to a single classifier and reduce the variance caused by a single classifier.

In this study, inspired by the success of ensemble classifier in the other fields, we proposed an ensemble classifier for protein remote homology detection, called SVM-Ensemble, which combined three state-of-the-art discriminative methods with a weighted voting strategy. The three basic classifiers SVM-Kmer, SVM-ACC, and SVM-SC-PseAAC were constructed with Kmer, auto-cross covariance (ACC), and series correlation pseudo amino acid composition (SC-PseAAC), respectively. Experimental results on a widely used benchmark dataset [5] showed that SVM-Ensemble can obviously improve the predictive performance by combining various features. Moreover, SVM-Ensemble achieved an average ROC score of 0.945, outperforming the other start-of-the-art methods, indicating that it would be a useful computational tool for protein remote homology detection.

## 2. Materials and Methods

### 2.1. Benchmark Dataset

A widely used superfamily benchmark [5] was used to evaluate the performance of our method for protein remote homology detection. The classification problem definition and benchmark dataset are available at http://noble.gs.washington.edu/proj/svm-pairwise/. The same dataset has been used in a number of earlier studies [26, 47–50], allowing us to perform direct comparisons to the relative performance.

The benchmark contains 54 families and 4352 proteins, which are derived from the SCOP database with version 1.53 and the similarities between any two sequences are less than *E*-value of 10^−25^. Remote homology detection can be treated as a superfamily classification problem. For each family, the proteins within the family were regarded as positive test samples, and the proteins outside the family but within the same superfamily were taken as positive training samples. Negative samples were selected from outside of the fold and split into training and testing sets. This process was repeated until each family had been tested. This yielded 54 families with at least 10 positive training examples and 5 positive test examples.

### 2.2. Profile-Based Protein Representation

Although some methods have achieved certain degree of success only by using amino acid sequence information, their performance is not satisfying. Recent studies demonstrated that the methods over profile-based protein sequences would show better performance because a profile is richer than an individual sequence as far as the evolutionary information is concerned [50, 53].

The frequency profile *𝕄* for protein **P** with *L* amino acids can be represented as

(1) $$\begin{array}{c} M=\left[\begin{array}{cccc} m_{1,1} & m_{1,2} & \cdots & m_{1,L}\\ m_{2,1} & m_{2,2} & \cdots & m_{2,L}\\ \vdots & \vdots & \vdots & \vdots \\ m_{20,1} & m_{20,2} & \cdots & m_{20,L} \end{array}\right], \end{array}$$

where *m*_*i*,*j*_  (0 ≤ *m*_*i*,*j*_ ≤ 1) is the target frequency which reflects the probability of amino acid *i* (*i* = 1,2,…, 20) occurring at the sequence position *j* (*j* = 1,2,…, *L*) in protein **P** during evolutionary processes. For each column in *𝕄*, the elements add up to 1. Each column can therefore be regarded as an independent multinomial distribution. The target frequency was calculated from the multiple sequence alignments generated by running PSI-BLAST [11] against the NCBI's NR with default parameters except that the number of iterations was set at 10 in the current study. The details of how to build a frequency profile can be found in [50].

Given the frequency profile *𝕄* for protein **P**, we can find the amino acid with maximum frequency in each column of *𝕄*. These amino acids are combined to produce the profile-based protein representation. In a frequency profile *𝕄*, the target frequencies reflect the probabilities of the corresponding amino acids appearing in the specific sequence positions. The higher the frequency is, the more likely the corresponding amino acid occurs. Thus, the produced profile-based protein sequence contains evolutionary information in the frequency profile. We convert the frequency profiles into a series of profile-based proteins. The existing sequence-based methods can therefore be directly performed on the protein representations for further processing.

### 2.3. Feature Vector Representations for Protein Sequences

In this study, three kinds of features have been employed to construct the SVM-Ensemble predictor, including Kmer, auto-cross covariance (ACC), and series correlation pseudo amino acid composition (SC-PseAAC).

Suppose a protein sequence **P** with *L* amino acid residues can be represented as

(2) $$\begin{array}{c} P=R_{1}R_{2}R_{3}R_{4}R_{5}R_{6}\cdots R_{L}, \end{array}$$

where *R*_*i*_ represents the amino acid residue at the sequence position *i*, such that *R*_1_ represents the amino acid residue at the sequence position 1 and *R*_2_ represents the amino acid residue at position 2 and so on. The three used representation methods can be described as follows.

#### 2.3.1. Kmer

Kmer [56] is the simplest approach to represent the proteins, in which the protein sequences are represented as the occurrence frequencies of *k* neighboring amino acids.

#### 2.3.2. Auto-Cross Covariance (ACC)

ACC transformation [60–62] is to build two signal sequences and then calculate the correlation between them. ACC results in two kinds of variables: autocovariance (AC) transformation and cross covariance (CC) transformation. AC variable measures the correlation of the same property between two residues separated by a distance of lag along the sequence. CC variable measures the correlation of two different properties between two residues separated by lag along the sequence.

*Autocovariance (AC) Transformation*. Given a protein sequence **P** in (2), the AC variable can be calculated by

(3) $$\begin{array}{c} AC\left(\;u,lag\;\right)=\overset{L-lag}{\underset{i=1}{\sum }}\frac{\left(P_{u}\left(R_{i}\right)-{\overset{-}{P}}_{u}\right)\left(P_{u}\left(R_{i+lag}\right)-{\overset{-}{P}}_{u}\right)}{L-lag}, \end{array}$$

where *u* is a physicochemical index, *L* is the length of the protein sequence, *P*_*u*_(*R*_*i*_) means the numerical value of the physicochemical index *u* for the amino acid *R*_*i*_, and ${\overset{-}{P}}_{u}$ is the average value for physicochemical index *u* along the whole sequence:

(4) $$\begin{array}{c} {\overset{-}{P}}_{u}=\overset{L}{\underset{j=1}{\sum }}\frac{P_{u}\left(R_{j}\right)}{L}. \end{array}$$

In such a way, the length of AC feature vector is *N∗*LAG, where *N* is the number of physicochemical indices. LAG is the maximum of lag (lag = 1,2,…, LAG).

*Cross Covariance (CC) Transformation*. Given a protein sequence **P** in (2), the CC variable can be calculated by

(5) CCu1,u2,lag=∑i=1L−lagPu1Ri−P−u1Pu2Ri+lag−P−u2L−lag,

where *u*_1_, *u*_2_ are two different physicochemical indices, *L* is the length of the protein sequence, and *P*_*u*_1__(*R*_*i*_), *P*_*u*_2__(*R*_*i*+lag_) are the numerical value of the physicochemical indices *u*_1_, *u*_2_ for the amino acids *R*_*i*_, *R*_*i*+lag_. ${\overset{-}{P}}_{u_{1}}$, ${\overset{-}{P}}_{u_{2}}$ are the average value for physicochemical index values *u*_1_, *u*_2_ along the whole sequence and they can be calculated by (4).

In such way, the length of the CC feature vector is *N∗*(*N* − 1)*∗*LAG, where *N* is the number of physicochemical indices. LAG is the maximum of lag (lag = 1,2,…, LAG).

Therefore, the length of the ACC feature vector is *N∗N∗*LAG. In current implementation, three physicochemical properties were employed, including hydrophobicity, hydrophilicity, and mass (see Table S1 in Supplementary file, available online at http://dx.doi.org/10.1155/2016/5813645) extracted from AAindex [57, 63].

#### 2.3.3. Series Correlation Pseudo Amino Acid Composition (SC-PseAAC)

SC-PseAAC [64] is an approach incorporating the contiguous local sequence-order information and the global sequence-order information into the feature vector of the protein sequence. Given a protein sequence **P** in (2), the SC-PseAAC [64] feature vector of **P** is defined:
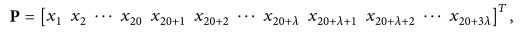
(6)where

(7) $$\begin{array}{c} x_{u}=\left\{\begin{array}{cc} \frac{f_{u}}{\sum_{i=1}^{20}f_{i}+w\sum_{j=1}^{2\lambda }\tau_{j}} & \left(1\leq u\leq 20\right)\\ \frac{w\tau_{u-20}}{\sum_{i=1}^{20}f_{i}+w\sum_{j=1}^{2\lambda }\tau_{j}} & \left(20+1\leq u\leq 20+3\lambda \right), \end{array}\right. \end{array}$$

where *f*_*i*_ (*i* = 1,2,…, 20) is the normalized occurrence frequency of the 20 native amino acids in the protein **P**; the parameter *λ* is an integer, representing the highest counted rank (or tier) of the correlation along a protein sequence; *w* is the weight factor ranging from 0 to 1; and *τ*_*j*_ is the *j*-tier sequence-correlation factor that reflects the sequence-order correlation between all of the most contiguous residues along a protein sequence, which is defined as

(8) $$\begin{array}{c} \tau_{1}=\frac{1}{L-1}\overset{L-1}{\underset{i=1}{\sum }}H_{i,i+1}^{1}\\ \tau_{2}=\frac{1}{L-1}\overset{L-1}{\underset{i=1}{\sum }}H_{i,i+1}^{2}\\ \tau_{3}=\frac{1}{L-1}\overset{L-1}{\underset{i=1}{\sum }}M_{i,i+1}\\ \vdots \\ \tau_{3\lambda -2}=\frac{1}{L-\lambda }\overset{L-\lambda }{\underset{i=1}{\sum }}H_{i,i+\lambda }^{1}\\ \tau_{3\lambda -1}=\frac{1}{L-\lambda }\overset{L-\lambda }{\underset{i=1}{\sum }}H_{i,i+\lambda }^{2}\\ \tau_{3\lambda }=\frac{1}{L-\lambda }\overset{L-\lambda }{\underset{i=1}{\sum }}M_{i,i+\lambda }\\ \;\left(\lambda <L-1\right), \end{array}$$

where *H*_*i*,*j*_^1^, *H*_*i*,*j*_^2^, and *M*_*i*,*j*_ are the hydrophobicity, hydrophilicity, and mass correlation functions given by

(9) Hi,j1=h^1Ri·h^1RjHi,j2=h^2Ri·h^2RjMi,j=m^Ri·m^Rj,

where ${\hat{h}}^{1}\left(R_{i}\right)$, ${\hat{h}}^{2}\left(R_{i}\right)$, and $\hat{m}\left(R_{i}\right)$ are the substituting values of hydrophobicity, hydrophilicity, and mass values for amino acid *R*_*i*_. They are all subjected to a standard conversion as described by the following equation:

(10) h^1Ri=h1Ri−∑k=120h1Rk/20∑u=120h1Ru−∑k=120h1Rk/202/20h^2Ri=h2Ri−∑k=120h2Rk/20∑u=120h2Ru−∑k=120h2Rk/202/20m^Ri=mRi−∑k=120mRk/20∑u=120mRu−∑k=120mRk/202/20,

where we use *ℝ*_*i*_ (*i* = 1,2,…, 20) to represent the 20 native amino acids. The symbols *h* ^1^(*R*_*i*_), *h* ^2^(*R*_*i*_), and *m*(*R*_*i*_) represent the original hydrophobicity, hydrophilicity, and mass values (see Table S1 in Supplementary file) of the amino acid *R*_*i*_.

These aforementioned features can be generated by a web-server called Pse-in-one [56], which can be used to generate the desired feature vectors for protein/peptide and DNA/RNA sequences according to the need of user's studies. It covers a total of 28 different modes, of which 14 are for DNA sequences, 6 are for RNA sequences, and 8 are for protein sequences.

### 2.4. Support Vector Machine

Support vector machine (SVM) is a supervised machine learning technique for classification task based on statistical theory [65, 66]. Given a set of fixed length training vectors with labels (positive and negative input samples), SVM can learn a linear decision boundary to discriminate the two classes. The result is a linear classification rule that can be used to classify new test samples. When the samples are linearly nonseparable, the kernel function can be used to map the samples to a high-order feature space in which the optimal hyper plane as decision boundary can be found. SVM has exhibited excellent performance in practice [54, 58, 67–73] and has a strong theoretical foundation of statistical learning.

In this study, the publicly available Gist SVM package (http://www.chibi.ubc.ca/gist/) is employed. The SVM parameters are used by default of the Gist Package except that the kernel function is set as radial basis function.

### 2.5. Ensemble Classifier

The ensemble classifier is able to learn a more expressive concept in classification compared to a single classifier and reduces the variance caused by a single classifier. Therefore, it was employed in many fields and achieved great success [36, 37].

**Figure 1:**
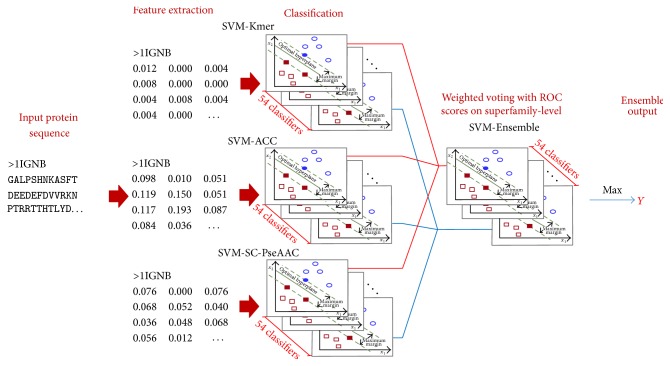
Flowchart to show how the ensemble classifier is formed by combining three basic classifiers on superfamily-level. The ensemble strategy is first employed on superfamily-level, and then the query protein **P** is predicted belonging to the superfamily type with which its score is the highest.

In this paper, we proposed a weighted voting strategy for protein remote homology detection, as shown in Figure 1. The ensemble framework of SVM-Ensemble was constructed by combining SVM-Kmer, SVM-ACC, and SVM-SC-PseAAC with weighted factors. The processing can be formulated as below.

Suppose the ensemble classifier is expressed by

(11) C=max⁡CS1,CS2,…,CS54

(12) CSj=C1Sj⊕C2Sj⊕C3Sj,

where *ℂ*_*iS*_*j*__ represents the *i*th basic SVM classifier on superfamily *S*_*j*_  (1 ≤ *j* ≤ 54). That is, *ℂ*_1*S*_1__ represents the classifier SVM-Kmer that operates on the superfamily *S*_1_, *ℂ*_2*S*_1__ represents the classifier SVM-ACC that operates on superfamily *S*_1_, and *ℂ*_3*S*_1__ represents the classifier SVM-SC-PseAAC that operates on superfamily *S*_1_. *ℂ*_*S*_*j*__ is the average performance of three basic classifiers on superfamily *S*_*j*_ with weighted voting strategy. In (12), the symbol ⊕ denotes the weighted voting operator.

The three basic classifiers can be combined by using the following equation:

(13) $$\begin{array}{c} C_{S_{j}}=\overset{3}{\underset{i=1}{\sum }}w_{iS_{j}}C_{iS_{j}}\left(\;P,S_{j}\;\right)\;\left(1\leq j\leq 54\right), \end{array}$$

where *ℂ*_*iS*_*j*__(**P**, *S*_*j*_) is the belief function or supporting degree for **P** belonging to *S*_*j*_ predicted by the *i*th basic classifier and *w*_*iS*_*j*__ is the weighted factor assigned with the average ROC score of the *i*th basic classifier on superfamily *S*_*j*_.

### 2.6. Performance Metrics for Evaluation

We evaluated the performance of different methods by employing the receiver operating characteristic (ROC) scores [55, 74–78]. Because the test sets have more negative than positive samples, simply measuring error-rates will not give a good evaluation of the performance. For the case, the best way to evaluate the trade-off between the specificity and sensitivity is to use ROC score. ROC score is the normalized area under a curve that is plotted with true positives as a function of false positives for varying classification thresholds. ROC score of 1 indicates a perfect separation of positive samples from negative samples, whereas ROC score of 0.5 denotes that random separation. ROC50 score is the area under the ROC curve up to the first 50 false positives.

## 3. Results and Discussion

### 3.1. The Influence of Parameters on the Predictive Performance of Basic Predictors

**Figure 2:**
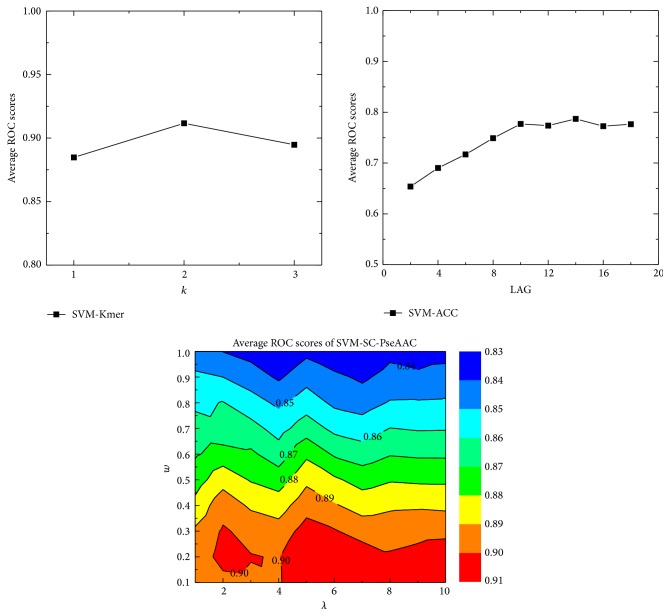
The performance of three basic predictors with all parameter combinations. *k* value of 2 and the LAG value of 14 were used in SVM-Kmer and SVM-ACC. SVM-SC-PseAAC achieves the best performance with *λ* = 5 and *w* = 0.2. Parameter *w* is mainly impact factor. However, parameter *λ* has minor impact on the performance.

There are several parameters for each basic predictor, which should be optimized. For more information of these parameters, please refer to Materials and Method. In this study, we optimized them by using grid search. The influence of these parameters on the performance was shown in Figure 2, and the optimized values of the parameters and their results were shown in Table 1, from which we can see that SVM-Kmer achieved the best performance, followed by SVM-SC-PseAAC.

### 3.2. Performance of Ensemble Classifier Based on Various Feature Combinations with Weighted Voting Strategy

As discussed above, predictors based on different feature sets showed different performance. In this study, in order to further improve the performance of protein remote homology detection, we employed an ensemble learning approach to combine various predictors. The performance of ensemble classifier combined various feature combinations was shown in Table 2. The best performance (ROC = 0.943, ROC50 = 0.744) can be achieved with the combination of all the three basic predictors and obviously outperformed all the three basic predictors in terms of both ROC score and ROC50 score. These results were not surprising. The three basic predictors were based on different features, and their predictive results are complementary. The performance can be improved by combining them with an ensemble learning method.

### 3.3. Feature Analysis for Discriminative Power

To further study the discriminative power of features in the three basic predictors, we employed a feature extraction method, called principal component analysis (PCA) [79], to calculate the discriminative weight vectors in the feature space. The process of PCA for extracting significant features can be found in [32, 80].

For each basic predictor, the top 10 most discriminative features in the feature space were shown in Table 3, from which we can see that, for the Kmer features, six of the most discriminative features contain the amino acid *M*, indicating the importance of this amino acid. For ACC features, the hydrophobicity (*h* ^1^) has important impact on the feature discrimination. For SC-PseAAC features, the amino acid *M* has the most discriminative power and features with small *λ* value are more important. Both ACC and SC-PseAAC features with strong discriminative power incorporate the sequence-order effects. These three kinds of features consider both sequence composition and sequence order effects. Therefore, SVM-Ensemble can further improve the performance by combining them in an ensemble learning approach.

### 3.4. Comparison with Other Related Predictors

Some state-of-the-art methods for protein remote homology detection were selected to compare with the proposed SVM-Ensemble. SVM-Pairwise [5] represents each protein as a vector of pairwise similarities to all proteins in the training set. The kernel of SVM-LA [26] measures the similarity between a pair of proteins by taking into account all the optimal local alignment scores with gaps between all possible subsequences. Mismatch kernel [28] is calculated based on occurrences of (*k*, *m*)-patterns in the data. Monomer-dist [47] constructs the feature vectors by the occurrences of short oligomers. SVM-DR is based on the distance-pairs; PseAACIndex is based on the pseudo amino acid composition (PseAAC). disPseAAC constructs the feature vectors by combining the occurrences of amino acid pairs within Chou's pseudo amino acid composition.

Experimental results of various methods on SCOP 1.53 benchmark dataset were shown in Table 4. The SVM-Ensemble achieved the best performance, indicating that it is correct to combine different predictors via an ensemble learning approach.

## 4. Conclusions

In this study, we have proposed an ensemble classifier for protein remote homology detection, called SVM-Ensemble. It was constructed by combining three basic classifiers with a weighted voting strategy. Experimental results on a widely used benchmark dataset showed that our method achieved ROC score of 0.943, which is obviously better than the three basic predictors, including SVM-Kmer, SVM-ACC, and SVM-SC-PseAAC. Compared with some other state-of-the-art methods, the SVM-Ensemble achieved the best performance. Furthermore, by analyzing the discriminative power of these features, some interesting patterns were discovered.

For the future work, more effective features and machine learning techniques will be explored. And evolutionary computation [81], the ensemble learning techniques, and neural-like computing models [82–87] would be applied to other bioinformatics problems, such as gene-disease relationship prediction [52, 88–92] and DNA motif identification [59, 93].

## Supplementary Material

The amino acids physicochemical indices and corresponding values for hydrophobicity, hydrophilicity and mass.

## Figures and Tables

**Table 1:** The performance of three basic predictors with optimal parameters on benchmark dataset.

Methods	Optimal parameters	ROC^[a]^	ROC50^[a]^
SVM-Kmer	*k* = 2	0.912	0.785
SVM-ACC	LAG = 14	0.787	0.483
SVM-SC-PseAAC	*λ* = 5, *w* = 0.2	0.911	0.657

^[a]^Average ROC and ROC50 scores.

**Table 2:** Performance of ensemble classifier combining various predictors with weighted voting. The best performance was achieved by combining SVM-Kmer, SVM-ACC, and SVM-SC-PseAAC. The symbol ⊕ denotes the weighted voting operator.

Ensemble methods with superfamily-level strategy	ROC^[a]^	ROC50^[a]^
SVM-Kmer ⊕ SVM-ACC	0.929	0.767
SVM-Kmer ⊕ SVM-SC-PseAAC	0.937	0.715
SVM-ACC ⊕ SVM-SC-PseAAC	0.922	0.691
SVM-Kmer ⊕ SVM-ACC ⊕ SVM-SC-PseAAC	**0.943**	**0.744**

^[a]^Average ROC and ROC50 scores.

**Table 3:** Top 10 most discriminative features in three feature spaces. These features describe the characteristics of proteins from various perspectives.

Rank	Kmer	ACC	SC-PseAAC
1	MH	CC_*h*^1^*h*^2^,lag=9_	*M*
2	WC	AC_*h*^1^,lag=5_	*Y*
3	IM	CC_*h*^1^*h*^2^,lag=8_	*τ*_*h*^2^,*λ*=1_
4	MC	AC_*h*^1^,lag=4_	*τ*_*h*^2^,*λ*=4_
5	MY	CC_*h*^1^*h*^2^,lag=7_	*H*
6	VM	AC_*h*^1^,lag=14_	*τ*_*h*^1^,*λ*=4_
7	YW	AC_*m*,lag=13_	*G*
8	YR	CC_*h*^1^*m*,lag=13_	*τ*_*h*^1^,*λ*=1_
9	HW	CC_*h*^1^*h*^2^,lag=10_	*τ*_*m*,*λ*=1_
10	MQ	AC_*h*^1^,lag=8_	*τ*_*m*,*λ*=3_

Note: the subscript indexes in ACC features and SC-PseAAC features mean hydrophobicity (*h* ^1^), hydrophilicity (*h* ^2^), and mass (*m*).

**Table 4:** Performance comparison of different methods on the benchmark dataset.

Methods	ROC^[a]^	ROC50^[a]^	Source
SVM-Ensemble	0.943	0.744	This study

SVM-Pairwise	0.896	0.464	Liao and Noble, 2003 [5]
SVM-LA (*β* = 0.5)	0.925	0.649	Saigo et al., 2004 [26]
Mismatch	0.925	0.649	Leslie et al., 2004 [28]

Monomer-dist	0.919	0.508	Lingner and Meinicke, 2006 [47]
SVM-WCM	0.904	0.445	Lingner and Meinicke, 2008 [51]

SVM-Ngram-LSA	0.859	0.628	Dong et al., 2006 [48]
SVM-Pattern-LSA	0.879	0.626	Dong et al., 2006 [48]
SVM-Motif-LSA	0.859	0.628	Dong et al., 2006 [48]
SVM-Top-n-gram-combine-LSA	0.939	0.767	Liu et al., 2008 [4]

PseAACIndex (*λ* = 5)	0.880	0.620	Liu et al., 2013 [31, 52]
PseAACIndex-Profile (*λ* = 5)	0.922	0.712	Liu et al., 2013 [31, 52]
SVM-DR	0.919	0.715	Liu et al., 2014 [50, 53–55]
disPseAAC	0.922	0.721	Liu et al., 2015 [2, 32, 44, 45, 56–59]

^[a]^Average ROC and ROC50 scores.

## References

[1] Bork P., Koonin E. V. (1998). Predicting functions from protein sequences—where are the bottlenecks?. *Nature Genetics*.

[2] Liu B., Chen J., Wang X. (2015). Application of learning to rank to protein remote homology detection. *Bioinformatics*.

[3] Rost B. (1999). Twilight zone of protein sequence alignments. *Protein Engineering*.

[4] Liu B., Wang X., Lin L., Dong Q., Wang X. (2008). A discriminative method for protein remote homology detection and fold recognition combining Top-n-grams and latent semantic analysis. *BMC Bioinformatics*.

[5] Liao L., Noble W. S. (2003). Combining pairwise sequence similarity and support vector machines for detecting remote protein evolutionary and structural relationships. *Journal of Computational Biology*.

[6] Needleman S. B., Wunsch C. D. (1970). A general method applicable to the search for similarities in the amino acid sequence of two proteins. *Journal of Molecular Biology*.

[7] Zou Q., Hu Q., Guo M., Wang G. (2015). HAlign: fast multiple similar DNA/RNA sequence alignment based on the centre star strategy. *Bioinformatics*.

[8] Smith T. F., Waterman M. S. (1981). Identification of common molecular subsequences. *Journal of Molecular Biology*.

[9] Altschul S. F., Gish W., Miller W., Myers E. W., Lipman D. J. (1990). Basic local alignment search tool. *Journal of Molecular Biology*.

[10] Pearson W. R. (1991). Searching protein sequence libraries: comparison of the sensitivity and selectivity of the Smith-Waterman and FASTA algorithms. *Genomics*.

[11] Altschul S. F., Madden T. L., Schäffer A. A. (1997). Gapped BLAST and PSI-BLAST: a new generation of protein database search programs. *Nucleic Acids Research*.

[12] Liu B., Wang X., Lin L., Dong Q., Wang X. (2009). Exploiting three kinds of interface propensities to identify protein binding sites. *Computational Biology and Chemistry*.

[13] Remmert M., Biegert A., Hauser A., Söding J. (2012). HHblits: lightning-fast iterative protein sequence searching by HMM-HMM alignment. *Nature Methods*.

[14] Eddy S. R. (1998). Profile hidden Markov models. *Bioinformatics*.

[15] Karplus K., Barrett C., Hughey R. (1998). Hidden Markov models for detecting remote protein homologies. *Bioinformatics*.

[16] Ding H., Lin H., Chen W. (2014). Prediction of protein structural classes based on feature selection technique. *Interdisciplinary Sciences: Computational Life Sciences*.

[17] Ding H., Liu L., Guo F.-B., Huang J., Lin H. (2011). Identify golgi protein types with modified mahalanobis discriminant algorithm and pseudo amino acid composition. *Protein and Peptide Letters*.

[18] Lin H., Liu W. X., He J., Liu X. H., Ding H., Chen W. (2015). Predicting cancerlectins by the optimal g-gap dipeptides. *Scientific Reports*.

[19] Liu B., Wang X., Chen Q., Dong Q., Lan X. (2012). Using amino acid physicochemical distance transformation for fast protein remote homology detection. *PLoS ONE*.

[20] Zhao X., Zou Q., Liu B., Liu X. (2014). Exploratory predicting protein folding model with random forest and hybrid features. *Current Proteomics*.

[21] Song L., Li D., Zeng X., Wu Y., Guo L., Zou Q. (2014). nDNA-prot: Identification of DNA-binding proteins based on unbalanced classification. *BMC Bioinformatics*.

[22] Lin C., Zou Y., Qin J. (2013). Hierarchical classification of protein folds using a novel ensemble classifier. *PLoS ONE*.

[23] Wei L., Liao M., Gao X., Zou Q. (2015). An improved protein structural classes prediction method by incorporating both sequence and structure information. *IEEE Transactions on Nanobioscience*.

[24] Wei L., Liao M., Gao X., Zou Q. (2015). Enhanced protein fold prediction method through a novel feature extraction technique. *IEEE Transactions on Nanobioscience*.

[25] Xu J., Zou Q., Xu R., Wang X., Chen Q. (2014). Using distances between Top-n-gram and residue pairs for protein remote homology detection. *BMC Bioinformatics*.

[26] Saigo H., Vert J.-P., Ueda N., Akutsu T. (2004). Protein homology detection using string alignment kernels. *Bioinformatics*.

[27] Ben-Hur A., Brutlag D. (2003). Remote homology detection: a motif based approach. *Bioinformatics*.

[28] Leslie C. S., Eskin E., Cohen A., Weston J., Noble W. S. (2004). Mismatch string kernels for discriminative protein classification. *Bioinformatics*.

[29] Rangwala H., Karypis G. (2005). Profile-based direct kernels for remote homology detection and fold recognition. *Bioinformatics*.

[30] Kuang R., Ie E., Wang K. (2005). Profile-based string kernels for remote homology detection and motif extraction. *Journal of Bioinformatics and Computational Biology*.

[31] Liu B., Wang X., Zou Q., Dong Q., Chen Q. (2013). Protein remote homology detection by combining chou's pseudo amino acid composition and profile-based protein representation. *Molecular Informatics*.

[32] Liu B., Chen J., Wang X. (2015). Protein remote homology detection by combining Chou’s distance-pair pseudo amino acid composition and principal component analysis. *Molecular Genetics and Genomics*.

[33] Zhang Y., Liu B., Dong Q., Jin V. X. (2011). An improved profile-level domain linker propensity index for protein domain boundary prediction. *Protein and Peptide Letters*.

[34] Dietterich T. G. (2000). Ensemble methods in machine learning. *Multiple Classifier Systems*.

[35] Lin C., Chen W., Qiu C., Wu Y., Krishnan S., Zou Q. (2014). LibD3C: ensemble classifiers with a clustering and dynamic selection strategy. *Neurocomputing*.

[36] Deng L., Guan J., Dong Q., Zhou S. (2009). Prediction of protein-protein interaction sites using an ensemble method. *BMC Bioinformatics*.

[37] Shen H.-B., Chou K.-C. (2006). Ensemble classifier for protein fold pattern recognition. *Bioinformatics*.

[38] Zou Q., Guo J., Ju Y., Wu M., Zeng X., Hong Z. (2015). Improving tRNAscan-SE annotation results via ensemble classifiers. *Molecular Informatics*.

[39] Liu B., Liu F., Fang L., Wang X., Chou K.-C. (2016). repRNA: a web server for generating various feature vectors of RNA sequences. *Molecular Genetics and Genomics*.

[40] Wang C. Y., Hu L., Guo M. Z., Liu X. Y., Zou Q. (2015). imDC: an ensemble learning method for imbalanced classification with miRNA data. *Genetics and Molecular Research*.

[41] Wei L., Liao M., Gao Y., Ji R., He Z., Zou Q. (2014). Improved and promising identification of human microRNAs by incorporatinga high-quality negative set. *IEEE/ACM Transactions on Computational Biology and Bioinformatics*.

[42] Chen J., Wang X., Liu B. (2016). iMiRNA-SSF: improving the identification of MicroRNA precursors by combining negative sets with different distributions. *Scientific Reports*.

[43] Liu B., Fang L., Liu F., Wang X., Chou K.-C. (2016). iMiRNA-PseDPC: microRNA precursor identification with a pseudo distance-pair composition approach. *Journal of Biomolecular Structure and Dynamics*.

[44] Liu B., Fang L., Wang S., Wang X., Li H., Chou K.-C. (2015). Identification of microRNA precursor with the degenerate K-tuple or Kmer strategy. *Journal of Theoretical Biology*.

[45] Liu B., Wang S., Wang X. (2015). DNA binding protein identification by combining pseudo amino acid composition and profile-based protein representation. *Scientific Reports*.

[46] Li L., Zhang Y., Zou L. (2012). An ensemble classifier for eukaryotic protein subcellular location prediction using gene ontology categories and amino acid hydrophobicity. *PLoS ONE*.

[47] Lingner T., Meinicke P. (2006). Remote homology detection based on oligomer distances. *Bioinformatics*.

[48] Dong Q.-W., Wang X.-L., Lin L. (2006). Application of latent semantic analysis to protein remote homology detection. *Bioinformatics*.

[49] Liao L., Noble W. S. Combining pairwise sequence similarity and support vector machines for remote protein homology detection.

[50] Liu B., Zhang D., Xu R. (2014). Combining evolutionary information extracted from frequency profiles with sequence-based kernels for protein remote homology detection. *Bioinformatics*.

[51] Lingner T., Meinicke P. (2008). Word correlation matrices for protein sequence analysis and remote homology detection. *BMC Bioinformatics*.

[52] Liu B., Yi J., Sv A. (2013). QChIPat: a quantitative method to identify distinct binding patterns for two biological ChIP-seq samples in different experimental conditions. *BMC Genomics*.

[53] Liu B., Liu B., Liu F., Wang X. (2014). Protein binding site prediction by combining hidden markov support vector machine and profile-based propensities. *The Scientific World Journal*.

[54] Liu W.-X., Deng E.-Z., Chen W., Lin H. (2014). Identifying the subfamilies of voltage-gated potassium channels using feature selection technique. *International Journal of Molecular Sciences*.

[55] Liu B., Xu J., Lan X. (2014). IDNA-Prot|dis: identifying DNA-binding proteins by incorporating amino acid distance-pairs and reduced alphabet profile into the general pseudo amino acid composition. *PLoS ONE*.

[56] Liu B., Liu F., Wang X., Chen J., Fang L., Chou K.-C. (2015). Pse-in-One: a web server for generating various modes of pseudo components of DNA, RNA, and protein sequences. *Nucleic Acids Research*.

[57] Liu B., Xu J., Fan S., Xu R., Zhou J., Wang X. (2015). PseDNA-Pro: DNA-binding protein identification by combining chou's PseAAC and Physicochemical distance transformation. *Molecular Informatics*.

[58] Liu B., Fang L., Chen J., Liu F., Wang X. (2015). MiRNA-dis: MicroRNA precursor identification based on distance structure status pairs. *Molecular BioSystems*.

[59] Liu B., Liu F., Fang L., Wang X., Chou K.-C. (2015). repDNA: a Python package to generate various modes of feature vectors for DNA sequences by incorporating user-defined physicochemical properties and sequence-order effects. *Bioinformatics*.

[60] Cao D.-S., Xu Q.-S., Liang Y.-Z. (2013). Propy: a tool to generate various modes of Chou's PseAAC. *Bioinformatics*.

[61] Dong Q., Zhou S., Guan J. (2009). A new taxonomy-based protein fold recognition approach based on autocross-covariance transformation. *Bioinformatics*.

[62] Liu X., Zhao L., Dong Q. (2011). Protein remote homology detection based on auto-cross covariance transformation. *Computers in Biology and Medicine*.

[63] Kawashima S., Kanehisa M. (2000). AAindex: amino acid index database. *Nucleic Acids Research*.

[64] Chou K.-C. (2005). Using amphiphilic pseudo amino acid composition to predict enzyme subfamily classes. *Bioinformatics*.

[65] Chang C.-C., Lin C.-J. (2011). LIBSVM: a Library for support vector machines. *ACM Transactions on Intelligent Systems and Technology*.

[66] Fang L., Liu F., Wang X., Chen J., Chou K.-C., Liu B. (2015). Identification of real microRNA precursors with a pseudo structure status composition approach. *PLoS ONE*.

[67] Ding H., Deng E.-Z., Yuan L.-F. (2014). ICTX-type: a sequence-based predictor for identifying the types of conotoxins in targeting ion channels. *BioMed Research International*.

[68] Ding H., Guo S.-H., Deng E.-Z. (2013). Prediction of Golgi-resident protein types by using feature selection technique. *Chemometrics and Intelligent Laboratory Systems*.

[69] Guo S.-H., Deng E.-Z., Xu L.-Q. (2014). INuc-PseKNC: a sequence-based predictor for predicting nucleosome positioning in genomes with pseudo k-tuple nucleotide composition. *Bioinformatics*.

[70] Lin H., Deng E.-Z., Ding H., Chen W., Chou K.-C. (2014). IPro54-PseKNC: a sequence-based predictor for identifying sigma-54 promoters in prokaryote with pseudo k-tuple nucleotide composition. *Nucleic Acids Research*.

[71] Lin H., Ding H., Guo F.-B., Zhang A.-Y., Huang J. (2008). Predicting subcellular localization of mycobacterial proteins by using Chou's pseudo amino acid composition. *Protein and Peptide Letters*.

[72] Yuan L.-F., Ding C., Guo S.-H., Ding H., Chen W., Lin H. (2013). Prediction of the types of ion channel-targeted conotoxins based on radial basis function network. *Toxicology in Vitro*.

[73] Liu B., Fang L., Long R., Lan X., Chou K.-C. (2016). iEnhancer-2L: a two-layer predictor for identifying enhancers and their strength by pseudo *k*-tuple nucleotide composition. *Bioinformaitcs*.

[74] Fawcett T. (2006). An introduction to ROC analysis. *Pattern Recognition Letters*.

[75] Ding H., Feng P.-M., Chen W., Lin H. (2014). Identification of bacteriophage virion proteins by the ANOVA feature selection and analysis. *Molecular BioSystems*.

[76] Ding H., Luo L., Lin H. (2009). Prediction of cell wall lytic enzymes using chou's amphiphilic pseudo amino acid composition. *Protein and Peptide Letters*.

[77] Liu B., Wang X., Lin L., Tang B., Dong Q., Wang X. (2009). Prediction of protein binding sites in protein structures using hidden Markov support vector machine. *BMC Bioinformatics*.

[78] Liu B., Fang L. (2016). Identification of microRNA precursor based on gapped *n*-tuple structure status composition kernel. *Computational Biology and Chemistry*.

[79] Wold S., Esbensen K., Geladi P. (1987). Principal component analysis. *Chemometrics and Intelligent Laboratory Systems*.

[80] Du Q.-S., Jiang Z.-Q., He W.-Z., Li D.-P., Chou K.-C. (2006). Amino acid principal component analysis (AAPCA) and its applications in protein structural class prediction. *Journal of Biomolecular Structure and Dynamics*.

[81] Zhang X., Tian Y., Jin Y. (2015). A knee point driven evolutionary algorithm for many-objective optimization. *IEEE Transactions on Evolutionary Computation*.

[82] Song T., Pan L. (2015). On the universality and non-universality of spiking neural P systems with rules on synapses. *IEEE Transactions on NanoBioscience*.

[83] Zeng X., Zhang X., Song T., Pan L. (2014). Spiking neural P systems with thresholds. *Neural Computation*.

[84] Chen X., Pérez-Jiménez M. J., Valencia-Cabrera L., Wang B., Zeng X. (2016). Computing with viruses. *Theoretical Computer Science*.

[85] Xiangxiang Zeng L. P., Pérez-Jiménez M. J. (2014). Small universal simple spiking neural P systems with weights. *Science China Information Sciences*.

[86] Zhang X., Pan L., Păun A. (2015). On the universality of axon P systems. *IEEE Transactions on Neural Networks and Learning Systems*.

[87] Zhang X., Liu Y., Luo B., Pan L. (2014). Computational power of tissue P systems for generating control languages. *Information Sciences*.

[88] Zeng X., Liao Y., liu Y., Zou Q. (2016). Prediction and validation of disease genes using HeteSim Scores. *IEEE/ACM Transactions on Computational Biology and Bioinformatics*.

[89] Zou Q., Li J., Hong Q. (2015). Prediction of microRNA-disease associations based on social network analysis methods. *BioMed Research International*.

[90] Zeng X., Zhang X., Zou Q. (2016). Integrative approaches for predicting microRNA function and prioritizing disease-related microRNA using biological interaction networks. *Briefings in Bioinformatics*.

[91] Zou Q., Li J., Song L., Zeng X., Wang G. (2016). Similarity computation strategies in the microRNA-disease network: a survey. *Briefings in Functional Genomics*.

[92] Chen H.-Z., Ouseph M. M., Li J. (2012). Canonical and atypical E2Fs regulate the mammalian endocycle. *Nature Cell Biology*.

[93] Wang X., Miao Y., Cheng M. (2014). Finding motifs in DNA sequences using low-dispersion sequences. *Journal of Computational Biology*.

