# Supplementary material for: Protein Remote Homology Detection Based on an Ensemble Learning Approach
Source: Biomed Res Int. 2016 May 8;2016:5813645. doi: 10.1155/2016/5813645 (PMC4875977; doi:10.1155/2016/5813645)
Supplement: Supplementary file 1 — The amino acids physicochemical indices and corresponding values for hydrophobicity, hydrophilicity and mass. [file 5813645.f1.pdf]

Table S1. The three kinds of physicochemical indices for amino acids and corresponding values.

Physicochemical properties	Hydrophobicity	Hydrophilicity	Mass
A	0.62	-0.5	71.079
R	-2.53	3	156.188
N	-0.78	0.2	114.104
D	-0.09	3	115.086
C	0.29	-1	103.145
Q	-0.85	0.2	128.131
E	-0.74	3	129.116
G	0.48	0	57.052
H	-0.4	-0.5	137.141
I	1.38	-1.8	113.160
L	1.53	-1.8	113.160
K	-1.5	3	128.170
M	0.64	-1.3	131.990
F	1.19	-2.5	147.177
P	0.12	0	97.177
S	-0.18	0.3	87.078
T	-0.05	-0.4	101.105
W	0.81	-3.4	186.123
Y	0.26	-2.3	163.176
V	1.8	-1.5	99.133
